# Impact of a combination of quantitative indices representing uptake intensity, shape, and asymmetry in DAT SPECT using machine learning: comparison of different volume of interest settings

**DOI:** 10.1186/s13550-019-0477-x

**Published:** 2019-01-28

**Authors:** Yu Iwabuchi, Tadaki Nakahara, Masashi Kameyama, Yoshitake Yamada, Masahiro Hashimoto, Yohji Matsusaka, Takashi Osada, Daisuke Ito, Hajime Tabuchi, Masahiro Jinzaki

**Affiliations:** 1 Department of Radiology, Keio University School of Medicine, 35 Shinanomachi, Shinjyuku-ku, Tokyo, 160-8582 Japan; 2 Department of Diagnostic Radiology, Tokyo Metropolitan Geriatric Hospital and Institute of Gerontology, 35-2 Sakaecho, Itabashi-ku, Tokyo, 173-0015 Japan; 3 Department of Neurology, Keio University School of Medicine, 35 Shinanomachi, Shinjyuku-ku, Tokyo, 160-8582 Japan; 4 Department of Neuropsychiatry, Keio University School of Medicine, 35 Shinanomachi, Shinjyuku-ku, Tokyo, 160-8582 Japan

**Keywords:** ^123^I-Ioflupane, ^123^I-FP-CIT, DAT SPECT, Parkinson’s syndrome, Support vector machine, Machine learning

## Abstract

**Background:**

We sought to assess the machine learning-based combined diagnostic accuracy of three types of quantitative indices obtained using dopamine transporter single-photon emission computed tomography (DAT SPECT)—specific binding ratio (SBR), putamen-to-caudate ratio (PCR)/fractal dimension (FD), and asymmetry index (AI)—for parkinsonian syndrome (PS). We also aimed to compare the effect of two different types of volume of interest (VOI) settings from commercially available software packages DaTQUANT (Q) and DaTView (V) on diagnostic accuracy.

**Methods:**

Seventy-one patients with PS and 40 without PS (NPS) were enrolled. Using SPECT images obtained from these patients, three quantitative indices were calculated at two different VOI settings each. SBR-Q, PCR-Q, and AI-Q were derived using the VOI settings from DaTQUANT, whereas SBR-V, FD-V, and AI-V were derived using those from DaTView. We compared the diagnostic value of these six indices for PS. We incorporated a support vector machine (SVM) classifier for assessing the combined accuracy of the three indices (SVM-Q: combination of SBR-Q, PCR-Q, and AI-Q; SVM-V: combination of SBR-V, FD-V, and AI-V). A Mann-Whitney *U* test and receiver-operating characteristics (ROC) analysis were used for statistical analyses.

**Results:**

ROC analyses demonstrated that the areas under the curve (AUC) for SBR-Q, PCR-Q, AI-Q, SBR-V, FD-V, and AI-V were 0.978, 0.837, 0.802, 0.906, 0.972, and 0.829, respectively. On comparing the corresponding quantitative indices between the two types of VOI settings, SBR-Q performed better than SBR-V (*p* = 0.006), whereas FD-V performed better than PCR-Q (*p* = 0.0003). No significant difference was observed between AI-Q and AI-V (*p* = 0.56). The AUCs for SVM-Q and SVM-V were 0.988 and 0.994, respectively; the two different VOI settings displayed no significant differences in terms of diagnostic accuracy (*p* = 0.48).

**Conclusion:**

The combination of the three indices obtained using the SVM classifier improved the diagnostic performance for PS; this performance did not differ based on the VOI settings and software used.

## Background

Two different types of volume of interest (VOI) settings have been proposed for routine clinical quantification of dopamine transporter single-photon emission computed tomography (DAT SPECT) results using VOI-based analyses. One of the two VOI settings uses a striatal VOI delineated to fit the striatum precisely. Some software packages, such as DaTQUANT (GE Healthcare, Little Chalfont, UK), apply this type of VOI setting [1]. This type of VOI setting needs registration to the normalized SPECT image because the shape and position of the striatum vary among individuals. This fixed-size VOI template can be divided to conform with the striatal structure parts, i.e., the caudate, the anterior putamen, and the posterior putamen, to enable the calculation of the putamen-to-caudate ratio (PCR) that represents the ratio between anterior and posterior striatal radioisotope (RI) uptake [1, 2]. Tossici-Bolt et al. proposed a large pentagonal prism-shaped VOI, which encompasses a wide area around the striatum (Southampton method), as an alternative setting [3]. Some software packages, such as DaTView (AZE, Tokyo, Japan), apply this type of VOI setting. This VOI can be set directly on the original image without normalization and has the merit of reducing the influence of a partial volume effect as it is large in size.

These two VOI settings can be used to evaluate three different types of quantitative indices that have complementary roles: the specific binding ratio (SBR), PCR/fractal dimension (FD), and asymmetry index (AI). The SBR index represents the amount of RI accumulation in the striatum, the PCR/FD index represents the distribution or the shape of the RI uptake, and the AI index represents the lateral difference between the right and left side RI uptake, respectively. Since the VOI from Tossici-Bolt et al. cannot be separated into anterior and posterior parts, the PCR index cannot be calculated using this VOI analysis. Therefore, another quantitative index that can replace the PCR index is required. Iwabuchi et al. proposed the FD index as a new quantitative index for assessing DAT SPECT results [4]. It can be calculated with the VOI from Tossici-Bolt et al. The FD index is used to evaluate the spatial heterogeneity or distribution of various types of medical images [5]. We consider that the PCR and FD indices have similar roles in assessing DAT SPECT as both indices represent the shape or the distribution of striatal RI accumulation.

In visual interpretations, a comprehensive evaluation of the three characteristic findings is important, i.e., the decline of striatal RI accumulation, the shape of RI accumulation, and the lateral difference in RI accumulation [6]. This is because the normal striatum shows symmetrical comma-shaped regions with high accumulation in both the caudate and putamen, while asymmetric dot-shaped patterns are observed in patients with Parkinsonian syndrome (PS). Therefore, we hypothesized that, even with quantitative assessments, a comprehensive evaluation with three characteristic indices, namely the SBR, PCR/FD, and AI, would be similarly important. Although several previous studies have demonstrated the utility of these quantitative indices individually [7–11] or assessed the combined accuracy of SBR and FD [4], to our knowledge, no study has reported the combined usefulness of the indices using the three features (SBR, PCR/FD, and AI).

Further, the VOI-setting style is thought to affect the diagnostic accuracy in quantitative assessment [12]. Although Shimizu et al. have compared the usefulness of the two types of VOI settings in distinguishing dementia with Lewy bodies (DLB) from Alzheimer’s disease (AD) [12]; they examined only the SBR index and did not examine other indices. Tossici-Bolt et al. and Yin et al. have demonstrated the influence of VOI settings on the SBR index itself [13, 14]. However, their studies did not assess the diagnostic accuracy for PS. To our knowledge, no study has assessed the effect of VOI settings on diagnostic accuracy for PS so far.

The purpose of this study was to assess the combined diagnostic accuracy of three types of DAT SPECT quantitative indices—SBR, PCR/FD, and AI—for PS using a support vector machine (SVM) classifier, and to compare the effect of two different types of VOI settings from commercially available software packages, i.e., DaTQUANT (Q) and DaTView (V).

## Materials and methods

### Patients

In this retrospective study, 183 consecutive patients who underwent DAT SPECT from February 2014 to August 2015 in our hospital were selected. Of these patients, 72 were excluded from the study because of the insufficiency of the image quality (*n* = 9) or clinical diagnosis (*n* = 63). The remaining 111 patients (median age, 68.5 years; range, 30–89 years; men/women, 62/49), including 71 patients with PS and 40 without PS (NPS), were included. Of the 71 patients with PS, 51 patients were diagnosed with PD based on the clinical diagnostic criteria of the UK Parkinson’s disease society brain bank, and the other 20 patients were diagnosed with atypical parkinsonian syndrome including clinical DLB (*n* = 11), progressive supranuclear palsy (PSP; *n* = 7), and multiple system atrophy (MSA; *n* = 2) on the basis of established diagnostic criteria [15–17].

The institutional review board of the hospital granted permission for this retrospective review of imaging and clinical data and waived the requirement for obtaining informed consent from the patients.

### SPECT acquisition and reconstruction

All SPECT images were acquired 3 h after the injection of ^123^I-Ioflupane (185 MBq) using the Discovery NM 630 or Discovery NM/CT 670 (GE Healthcare, Milwaukee, WI) with a FAN beam collimator. The imaging parameters were as follows: matrix size, 128 × 128; pixel size, 4.4 mm; slice thickness, 4.4 mm; and energy window, 159 keV ± 10%. The projection data acquired for 30 min were reconstructed on a Xeleris workstation (GE Healthcare). The ordered-subset expectation maximization (OSEM) method (iterations, 3; subset, 10) and a Butterworth filter setting with a critical frequency of 0.5 and power of 10.0 were applied for DaTView software. The OSEM (iterations, 2; subset, 10) and a Butterworth filter setting with a critical frequency of 0.6 and power of 10.0 were applied for DaTQUANT. Neither attenuation correction nor scatter correction was used. These settings were determined based on the manufacturer’s recommended settings.

### Quantitative indices calculated from VOI-based analyses

**Fig. 1:**
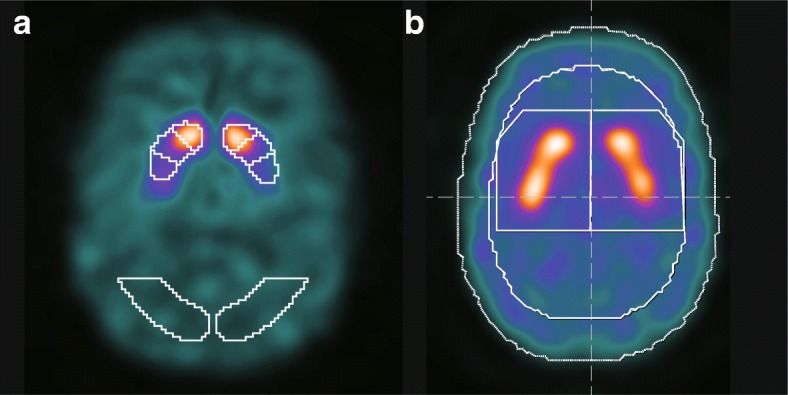
Two types of VOI settings using DaTQUANT and DaTView. **a** DaTQUANT uses separated VOIs delineated to fit to the striatum. The reference VOIs are set to the bilateral occipital lobes. **b** DaTView uses large pentagonal prism-shaped VOIs encompassing a wide area around the striatum. The reference VOI is set to the whole brain. Abbreviation: VOI, volume of interest

We used two commercially available software packages for VOI-based analyses: DaTQUANT (GE Healthcare, Little Chalfont, UK) and DaTView (AZE, Tokyo, Japan) (Fig. 1). These are representative of two different widespread VOI-based approaches. DaTView uses the VOI from Tossici-Bolt et al., while DaTQUANT uses a normalized VOI template. The VOI settings for the reference background also differ. DaTView applies the whole brain, except a region around the basal ganglia, as a reference region, while DaTQUANT applies the occipital lobe as a reference region [1, 3].

The three types of indices derived with the VOI setting of DaTQUANT were named SBR-Q, PCR-Q, and AI-Q, while the indices derived with the VOI setting of DaTView were named SBR-V, FD-V, and AI-V. Five indices (SBR-Q, PCR-Q, AI-Q, SBR-V, and AI-V), except for FD-V, can be computed automatically with DaTQUANT and DaTView software. SBR-Q is defined as the mean counts of the striatal VOI (background-subtracted) divided by the mean counts of the occipital lobe VOI, which simply represents the count ratio [1]. On the other hand, SBR-V is defined as the count concentration of the striatal VOI divided by the count concentration of the whole brain, which represents the concentration ratio [3]. Both the AI-Q and AI-V are defined as the difference between the SBR of both sides divided by the mean of the SBR of both sides. PCR-Q is defined as the mean counts of the putamen VOI divided by the mean counts of the caudate VOI [1]. The FD value can be calculated using the VOI setting from Tossici-Bolt et al. After setting 5-stage threshold values to delineate the striatum, the number of pixels obtained at each threshold was measured [4, 18–22]. The FD is calculated on the basis of the relationship between the threshold values and the number of pixels. For details, refer to a previous report [4].

### Support vector machine classifier

We used SVM classification, one of the classifiers for machine learning, to assess the combined diagnostic accuracy of the three quantitative indices. SVM classifiers have been used for classification analyses owing to their high accuracy and ability to deal with high dimensional data. The SVM algorithm computes the class separation boundaries to maximize the distance between the boundaries and the example points belonging to different classes [23, 24]. In this study, linear SVM classifiers were used and the separation hyperplanes were defined using the following formula (1), where **W** is the weight vector, *X* is the feature vector, and *b* is the bias.

1 $$ \mathbf{W}\times X+b=0 $$

We divided the dataset in such a way that 75% was used as a training dataset, and the remaining 25% was used as the testing data. This partitioning was stratified so that both datasets had the same class proportions. A tenfold cross validation was used for the training dataset to prevent overfitting [25]. The optimal **W** and *b* that best separate the PS and NPS groups were computed using the training dataset. Furthermore, we calculated the diagnostic accuracy of the test dataset using the trained SVM classifier. We incorporated the three types of indices as features for SVM classification: SVM-Q (derived using the SBR-Q, PCR-Q, and AI-Q) and SVM-V (derived using the SBR-V, FD-V, and AI-V). Each index was incorporated after standardization using the following formula (2), where *x* is the original feature vector, $\overline{x}$ is the mean of that feature vector, and *σ* is its standard deviation.

2 $$ {x}^{\prime }=\frac{x-\overline{x}}{\sigma } $$

We compared the diagnostic accuracy between SVM-Q and SVM-V to determine the combined diagnostic accuracy of the two types of VOI settings.

### Statistical analysis

Sex and age distribution were compared using the Fisher’s exact test or the *t* test. The Mann-Whitney *U* test was used to compare eight indices (SBR-Q, PCR-Q, AI-Q, SVM-Q, SBR-V, FD-V, AI-V, and SVM-V) between the PS and NPS groups. The receiver-operating characteristics (ROC) analysis of each index was performed to evaluate the AUC. We used the DeLong method to examine the difference between the two AUCs [26]. The sensitivity, specificity, positive predictive value (PPV), negative predictive value (NPV), and accuracy of each index were calculated using the optimal cutoff values determined based on the ROC curves. Differences with *p* values < 0.05 were considered statistically significant.

All statistical analyses were performed using the statistical package R (version 3.2.2; available as a free download from http://www.r-project.org) and SPSS software (version 14.0J; SPSS Inc., Chicago, IL).

## Results

### Demographic features

The characteristics of the selected patients are summarized in Table 1. There were no significant group differences in sex and age.

**Table 1:** Patient characteristics

	ALL	PS	NPS	*p* value
Number of cases	111	71	40	–
Age (year, mean ± SD)	69 ± 11.6	70 ± 10.2	66 ± 13.5	0.12^✱^
Men/women (*N*)	62/49	39/32	23/17	0.84^✝^

No significant differences were observed with respect to age and the sex ratio between the PS and NPS groups

*Abbreviations*: *PS* parkinsonian syndrome, *NPS* non-parkinsonian syndrome, *SD* standard deviation

^✱^*t* test

^✝^Fisher’s exact test

### Comparison among individual quantitative indices

**Fig. 2:**
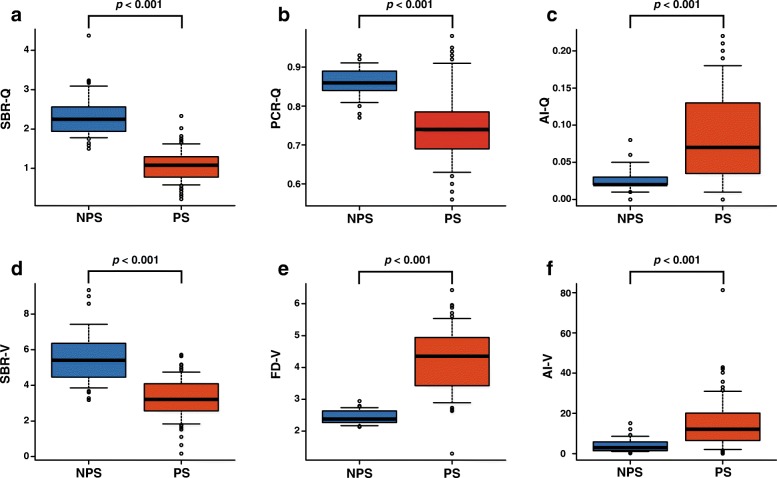
Box-and-whisker plots of the SBR-Q (**a**), PCR-Q (**b**), AI-Q (**c**), SBR-V (**d**), FD-V (**e**), and AI-V (**f**). There were significant differences in all six quantitative indices between NPS and PS, as assessed using the Mann-Whitney *U* test. Abbreviations: AI, asymmetry index; FD, fractal dimension; NPS, non-parkinsonian syndrome; PCR, putamen-to-caudate ratio; PS, parkinsonian syndrome; SBR, specific binding ratio

Figure 2 shows the box-and-whisker plots of the six quantitative indices used to differentiate between the PS and NPS groups. Significant differences were observed between the PS and NPS groups for every index (*p* < 0.001). The mean SBR-Q (Fig. 2a), SBR-V (Fig. 2d), and PCR-Q (Fig. 2b) of patients with NPS were significantly higher than those of patients in the PS group (2.32 ± 0.56 vs 1.07 ± 0.42, *p* < 0.001; 5.61 ± 1.51 vs 3.26 ± 1.15, *p* < 0.001; 0.86 ± 0.04 vs 0.75 ± 0.10, *p* < 0.001; respectively), while the mean AI-Q (Fig. 2c), AI-V (Fig. 2f), and FD-V (Fig. 2e) were significantly lower for the NPS than for the PS group (0.03 ± 0.02 vs 0.09 ± 0.06, *p* < 0.001; 3.94 ± 3.37 vs 15.2 ± 13.3, *p* < 0.001; 2.44 ± 0.21 vs 4.19 ± 1.06, *p* < 0.001; respectively).

**Fig. 3:**
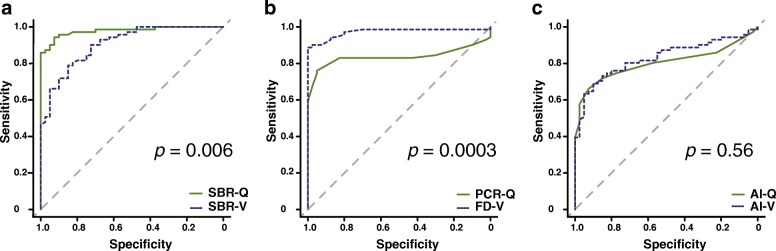
ROC analysis of the three types of indices. **a** The AUCs for SBR-Q and SBR-V were 0.978 and 0.906, respectively. **b** The AUCs for PCR-Q and FD-V were 0.837 and 0.972, respectively. **c** The AUCs for AI-Q and AI-V were 0.802 and 0.829, respectively. Abbreviations: AI, asymmetry index; AUC, area under the curve; FD, fractal dimension; PCR, putamen-to-caudate ratio; ROC, receiver-operating characteristics; SBR, specific binding ratio

The ROC analyses demonstrated that the AUCs for SBR-Q, PCR-Q, AI-Q, SBR-V, FD-V, and AI-V were 0.978, 0.837, 0.802, 0.906, 0.972, and 0.829, respectively (Fig. 3). On comparing the corresponding quantitative indices between the two software packages, SBR-Q performed better than SBR-V (*p* = 0.006), whereas FD-V performed better than PCR-Q (*p* = 0.0003). No significant difference was observed between AI-Q and AI-V (*p* = 0.56). Of the six indices, SBR-Q and FD-V showed better diagnostic performance than did other quantitative indices (SBR-Q, AUC = 0.978; FD-V, AUC = 0.972). Table 2 provides a summary of the sensitivity, specificity, PPV, NPV, and accuracy of the indices. The cutoff values for SBR-Q, PCR-Q, AI-Q, SBR-V, FD-V, and AI-V were 1.71, 0.81, 0.05, 4.16, 2.89, and 7.50, respectively.

**Table 2:** Sensitivity, specificity, PPV, NPV, and accuracy of the six indices

	Sensitivity (%)	Specificity (%)	PPV (%)	NPV (%)	Accuracy (%)
SBR-Q	94.4 (67/71)	92.5 (37/40)	95.7 (67/70)	90.2 (37/41)	93.7 (104/111)
SBR-V	78.9 (56/71)	85.0 (34/40)	90.3 (56/62)	69.4 (34/49)	81.1 (90/111)
PCR-Q	80.3 (57/71)	87.5 (35/40)	91.9 (57/62)	71.4 (35/49)	82.9 (92/111)
FD-V	90.1 (64/71)	97.5 (39/40)	98.5 (64/65)	84.8 (39/46)	92.8 (103/111)
AI-Q	71.8 (51/71)	85.0 (34/40)	89.5 (51/57)	63.0 (34/54)	76.6 (85/111)
AI-V	73.2 (52/71)	85.0 (34/40)	89.7 (52/58)	64.2 (34/53)	77.5 (86/111)

*Abbreviations*: *PPV* positive predictive value, *NPV* negative predictive value, *SBR* specific binding ratio, *PCR* putamen-to-caudate ratio, *FD* fractal dimension, *AI* asymmetry index

### Combined diagnostic accuracy of the three types of indices

**Fig. 4:**
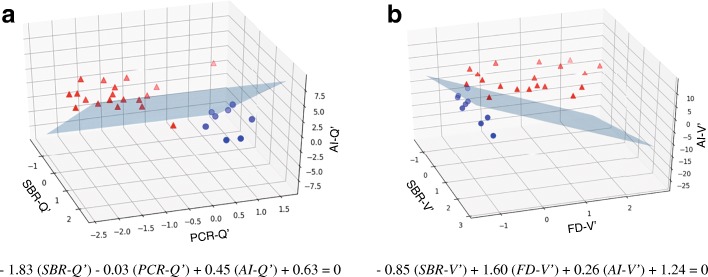
Scatter plots of the PS (red triangles) and NPS (blue circles) cases. The blue planes are the boundary surfaces determined by the SVM classifier. The apostrophe represents the means after standardization. **a** The SVM-Q computed with SBR-Q, PCR-Q, and AI-Q. **b** The SVM-V computed with SBR-V, FD-V, and AI-V. Abbreviations: AI, asymmetry index; FD, fractal dimension; NPS, non-parkinsonian syndrome; PCR, putamen-to-caudate ratio; PS, parkinsonian syndrome; SBR, specific binding ratio; SVM, support vector machine

**Fig. 5:**
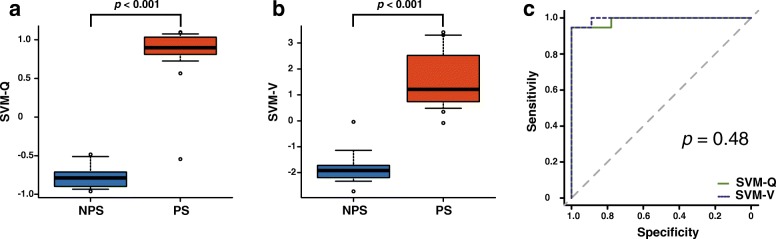
Box-and-whisker plots and ROC analysis of SVM-Q and SVM-V. There were significant differences between NPS and PS in both the SVM-Q (**a**) and SVM-V (**b**), as assessed using a Mann-Whitney *U* test. **c** The AUCs for the SVM-Q and SVM-V were 0.988 and 0.994, respectively. Abbreviations: AUC, area under the curve; NPS, non-parkinsonian syndrome; PS, parkinsonian syndrome; ROC, receiver-operating characteristics; SVM, support vector machine

Figure 4 shows the results of the combined diagnosis of the three types of indices using the SVM classifier. The blue planes are the boundary surface that divides the PS and NPS groups. Figure 5 shows the box-and-whisker plots and the ROC curve for SVM-Q and SVM-V. Significant differences were observed for both the SVM-Q (Fig. 5a) and SVM-V (Fig. 5b) results (− 0.76 ± 0.17 vs 0.84 ± 0.36, *p* < 0.001; − 1.78 ± 0.75 vs 1.56 ± 1.14, *p* < 0.001; respectively). The AUCs for SVM-Q and SVM-V were 0.988 and 0.994, respectively (Fig. 5c). No significant difference was observed between SVM-Q and SVM-V. The sensitivity, specificity, PPV, NPV, and accuracy are summarized in Table 3.

**Table 3:** Sensitivity, specificity, PPV, NPV, and accuracy of the SVM-Q and SVM-V

	Sensitivity (%)	Specificity (%)	PPV (%)	NPV (%)	Accuracy (%)
SVM-Q	94.7 (18/19)	100 (9/9)	100 (18/18)	90.0 (9/10)	96.4 (27/28)
SVM-V	94.7 (18/19)	100 (9/9)	100 (18/18)	90.0 (9/10)	96.4 (27/28)

*Abbreviations*: *PPV* positive predictive value, *NPV* negative predictive value, *SVM* support vector machine

## Discussion

The combined diagnostic accuracy of the three types of indices (AUC for SVM-Q = 0.988, AUC for SVM-V = 0.994) was numerically higher than the individual accuracies of the six indices (AUC = 0.802–0.978). This result suggests that the combination of the three features is useful, even for quantitative evaluation, similar to the visual interpretation described in the SNM practice guideline for DAT imaging [6]. Although several studies have demonstrated the utility of each index individually [7–11], this study is the first to quantitatively evaluate the combined usefulness of these indices. We believe that our proposed method involving a combination of the three characteristic indices can easily be used in routine clinical practice because these indices can be obtained simultaneously using simple VOI settings. This method can also reduce inter-operator variability as operators will only have to set the striatal VOI once; therefore, improvements in diagnostic accuracy may be achieved.

Our results demonstrate that no significant difference was observed when comparing the combined accuracies between two different types of VOI settings (*p* = 0.48). However, the SBR-Q delivered better accuracy than the SBR-V (*p* < 0.01). The method of Tossici-Bolt et al. can be influenced by the dilatation of the ventricles or the cerebral sulcus because this method uses a large VOI for the striatum [27, 28]. This might be why the accuracy of SBR-V was lower than that of SBR-Q in our study. In addition, FD-V showed better accuracy than PCR-Q (*p* < 0.001). PCR-Q simply represents the ratio between the anterior and posterior striatal accumulation, while FD-V can represent a change in the image through different stages using a calculation based on the threshold settings of the five stages [4]. We speculate that FD-V might be able to capture a change of distribution in more detail than PCR-Q. These results suggest that the combined use of the indices might compensate for individual differences between the corresponding indices of the two VOI settings.

To our knowledge, this is the first study to use the SVM classifier to combine the three types of indices (SBR, PCR/FD, and AI). Although several studies have evaluated the utility of SVM analysis in assessing DAT SPECT [29–32], no report has applied the three indices as features. Oliveira et al. used seven indices as features, including the SBR and PCR, as well as the length and volume of striatum uptake [29]. However, they did not include the AI index as a feature, and the seven indices used in their study cannot be obtained simultaneously using one VOI setting. Iwabuchi et al. previously assessed the combined accuracy of SBR and FD using an unsophisticated method of simply dividing SBR by FD, and did not use an SVM classifier to combine these quantitative indices, or include AI as a characteristic quantitative index [4].

This study had some limitations. First, the number of patients enrolled was relatively small. Additional studies with larger numbers of participants are needed to confirm our observations. Second, the diagnoses of PD and NPS were based on clinical observations, not pathology. Although this may have influenced our results, it is practically difficult to perform DAT SPECT and pathological examination at the same time. When autopsy is used as a reference standard, diagnostic discrepancies between DAT SPECT and autopsy findings can be noted, as the diagnosis can change with disease progression [33]. Third, we did not assess the effect of reconstruction parameters and scanner characterization. As indicated by Tossici-Bolt et al. [13], these factors would have differing effects on different VOI settings, and this matter requires further investigation.

## Conclusion

A combination of the three types of indices obtained using the SVM classifier improved the diagnostic performance for PS, and no significant difference was observed between the combined diagnostic performance of indices calculated using the VOI settings from two different software packages.

## Abbreviations

AD: Alzheimer’s disease
AI: Asymmetry index
AUC: The areas under the curve
DAT: Dopamine transporter
DLB: Dementia with Lewy bodies
FD: Fractal dimension
MSA: Multiple system atrophy
NPS: Non-parkinsonian syndrome
OSEM: Ordered-subset expectation maximization
PCR: Putamen-to-caudate ratio
PS: Parkinsonian syndrome
PSP: Progressive supranuclear palsy
RI: Radioisotope
ROC: Receiver-operating characteristics
SBR: Specific binding ratio
SPECT: Single-photon emission computed tomography
SVM: Support vector machine
VOI: Volume of interest
